# Morphometric Features and Microanatomy of the Lingual Filiform Papillae in the Wistar Rat

**DOI:** 10.3390/biology11060920

**Published:** 2022-06-16

**Authors:** Elena Huțanu, Aurel Damian, Viorel Miclăuș, Ioana A. Rațiu, Vasile Rus, Ion Vlasiuc, Adrian F. Gal

**Affiliations:** 1 Department of Anatomy, University of Agricultural Sciences and Veterinary Medicine Cluj-Napoca, 400372 Cluj-Napoca, Romania; elena.hutanu@usamvcluj.ro (E.H.); aurel.damian@usamvcluj.ro (A.D.); ion.vlasiuc@usamvcluj.ro (I.V.); 2 Department of Cell Biology, Histology and Embryology, Faculty of Veterinary Medicine, University of Agricultural Sciences and Veterinary Medicine Cluj-Napoca, 400372 Cluj-Napoca, Romania; vmiclaus@usamvcluj.ro (V.M.); vasile.rus@usamvcluj.ro (V.R.); adrian.gal@usamvcluj.ro (A.F.G.); 3 Department of Medical Sciences, Faculty of Medicine, University of Oradea, 410073 Oradea, Romania

**Keywords:** tongue, filiform papillae, rat, microanatomy

## Abstract

**Simple Summary:**

The tongue plays an important role in all animals but especially in mammals. It participates in food and water intake, as well as in some behavioral activities such as grooming. Its structure differs depending on the species. We aimed to provide a detailed macroscopic and microscopic description of the filiform papillae on the surface of rat tongue. We examined fragments on three regions of the tongue (tip, body, root) and observed an intensely keratinized epithelium on the whole surface of the tongue, with higher keratinization on the filiform papillae. We also identified differences in the density of the papillae dependent on the region examined, with the highest density being present on the back of the tongue. Additionally, we noted differences in the height of the filiform papillae, with the shortest being on the tip of the tongue and the tallest on the middle of the tongue. The unusual posteroanterior inclination of the filiform papillae from the tongue protuberance suggests they may play some other obscure roles compared to anteroposteriorly oriented filiform papillae. The study of macro and microanatomy in mammals is important since it helps in the assessment and understanding of anatomical and behavioral features of species.

**Abstract:**

The mammalian tongue plays a fundamental role in various physiological and behavioral activities. Significant morphological variations have been recorded in the tongue of several species. This study aims to obtain detailed histological and morphometric information about the filiform papillae on the surface of rat tongue. The tongues of five 10-month-old Wistar rats were utilized, which were later examined with a stereo-microscope. Fragments from the three regions of the tongue were collected for histological investigations. The tongue of the Wistar rat has an intensely keratinized stratified squamous epithelium, with the highest degree of keratinized epithelium covering the filiform papillae. The filiform papillae differ in density, with the highest density recorded on the posterior part of the lingual body and the lowest density on the protuberance. The shortest filiform papillae were observed on the apex of the tongue and the tallest on the anterior part of the lingual body. Interestingly, the orientation of the filiform papillae on the lingual protuberance was inclined posteroanteriorly, in the opposite way as compared to the papillae from all the other regions of the tongue. Histologically, a difference was recorded in the structure of the covering epithelium of the anterior vs. the posterior face of the filiform papillae.

## 1. Introduction

The tongue first appeared in amphibians as an adaptive structure to terrestrial food and as a necessity for adaptation to a wider range of habitats. The epithelium lining on the surface of the tongue is directly related to the environment in which the species lives. Frogs that live in water have a stratified cuboidal epithelium, but those that also live on land have a stratified squamous epithelium. The influence of the environment on the surface epithelium of the tongue is also found in the degree of keratinization. While freshwater frogs do not show signs of keratinization of the lingual epithelium, terrestrial and marine frogs have keratinized epitheliums [1].

Mammalian tongues play a key role in capturing and handling food, drinking water, swallowing, grooming, vocal modulation, and breastfeeding [2,3,4,5]. A variety of morphological structures of the mammalian tongue participate in the performance of these functions and are directly associated with dietary specializations and types of food, as well as with adaptations to different environmental conditions [1,6,7]. In order to fulfill these functions, the tongue presents significant morphological variations that seem to represent adaptations to the environmental conditions of each habitat. While in many species the tongue actively participates in food grip, there are species in which this function is less important. These include raccoons, sea otters, primates, and even humans, who use their limbs to bring food into their mouths. In the case of the human species, who largely consume cooked food that is softer, the keratinization processes of the dorsal surface of the tongue is significantly more discreet than in animals that consume harsher feed [1].

The mammalian tongue has some differences in the size, shape, presence, and characteristics of the lingual papillae and types of salivary glands. The differences between the number, type, and distribution of the existing papillae on the lingual surface are larger between animals belonging to different orders and families, but sometimes they can be species specific [8]. The lingual papillae are the fundamental structures involved in directing ingested fluids and food to all mammals, regardless of their age [9].

In most mammals, there are four types of lingual papillae: filiform, fungiform, foliate, and vallate or goblet-shaped papillae [5]. Functionally, lingual papillae are divided into two categories: mechanical papillae, including filiform, conical or lenticular papillae; and chemical or gustatory papillae, including fungiform, circumvallate, and foliate papillae [10]. There are exceptions to this rule in the case of herbivores that have only filiform, fungiform, and vallate papillae, but not foliated papillae [11]. Although the distribution, size, or number of lingual papillae are different between animals, their role is similar between herbivores and carnivores [10].

The filiform papillae are usually inclined anteroposteriorly, facilitating the retention of food on the dorsal surface of the tongue. In some species (e.g., cats), the filiform papillae are also used for grooming, the keratinization of those papillae being more pronounced in this case so that they can withstand greater mechanical stress than in the case of other animals [12]. The filiform papillae are present on the entire surface of the tongue and can have different subtypes and shapes [13]. In comparative studies, some authors have found both similarities and differences between the filiform papillae of different mammal species [14].

In order to obtain detailed information about the filiform papillae on the surface of the rat tongue, we set out to perform histological and morphometric investigations in the hope that the results obtained may serve as a basis for future research in the field of pathology and experimental medicine.

## 2. Materials and Methods

The biological material used in this study was five 10-month-old white Wistar rats. The study was approved by the Bioethics Committee of the University of Agricultural Sciences and Veterinary Medicine Cluj-Napoca, Romania (no. 292/22 November 2021). The tongue samples were harvested and examined with a stereo-microscope, during which photos were taken from all regions of the tongue. The density of the filiform papillae in each region of the tongue was determined by counting them from 14 square sections with a size of 1 cm. Fragments from three parts of the tongue—the tip, body and root—were collected for histological investigations. The samples were fixed for 7 days in 10% buffered formalin, dehydrated with increasing alcohol (70°, 96°, and absolute), clarified with 1-butanol, and embedded in paraffin. Longitudinal 5 μm thick sections were performed on a LEICA RM2125RT microtome and stained with Goldner’s trichrome method. The histological slides obtained were examined under an Olympus BX41 (Tokyo, Japan) microscope equipped with an Olympus E330 digital camera (Tokyo, Japan). Microscopic photographs were taken from all regions of the tongue, using the same lens to make the results comparable. For the morphometric aspects, the digital microscopy program AmScope v4.8.15934 (Amicroscope Ltd., Surrey, UK) was used.

## 3. Results and Discussion

**Figure 1:**
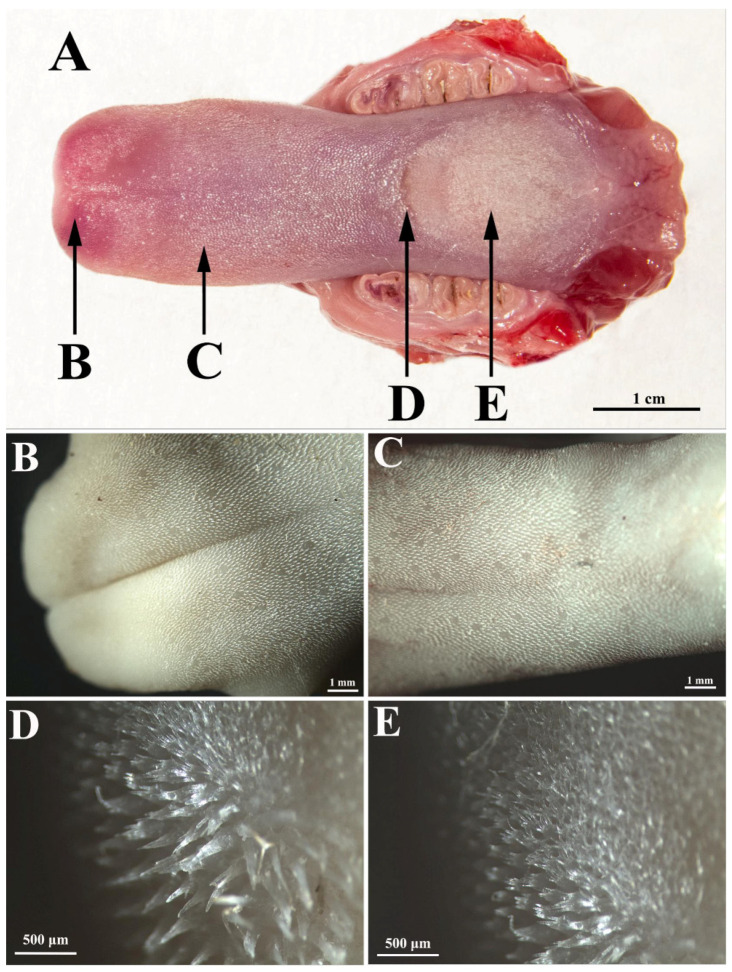
Gross features of the tongue of the white Wistar rat: (**A**) the organ as a whole that includes the apex, the anterior portion of the lingual body, the protuberance, and the posterior portion of the lingual body; (**B**) the lingual apex that includes the shortest filiform papillae of all the tongue, with a sharp tip and a pronounced anteroposterior inclination (claw appearance); (**C**) the anterior portion of the lingual body displaying slightly taller filiform papillae (vs. the apex) with an anteroposterior orientation and slightly lower density (vs. the apex); (**D**) tongue protuberance showing the tallest filiform papillae compared to all other regions of the tongue, with a posteroanterior-inclined conical tip; (**E**) the posterior part of the body of the tongue covered with anteroposteriorly inclined papillae that are slightly shorter vs. the ones from the protuberance.

The rat tongue consists of three regions (Figure 1A): the apex (*apex linguae*), the body (*corpus linguae*), and the root (*radix linguae*). The tongue of the white Wistar rat has an obvious protuberance, in front of which there is a lingual fossa, and at the apex there is a median groove.

In the white Wistar rat, the dorsal surface of the tongue is covered with filiform papillae on all three areas, namely the tip, body, and root of the tongue. However, there are large differences between the three areas in terms of the density, height, and appearance of the filiform papillae (Table 1).

On the lingual apex, the filiform papillae are relatively short (Figure 1A,B) with a sharp tip and a pronounced anteroposterior inclination (claw appearance).

**Figure 2:**
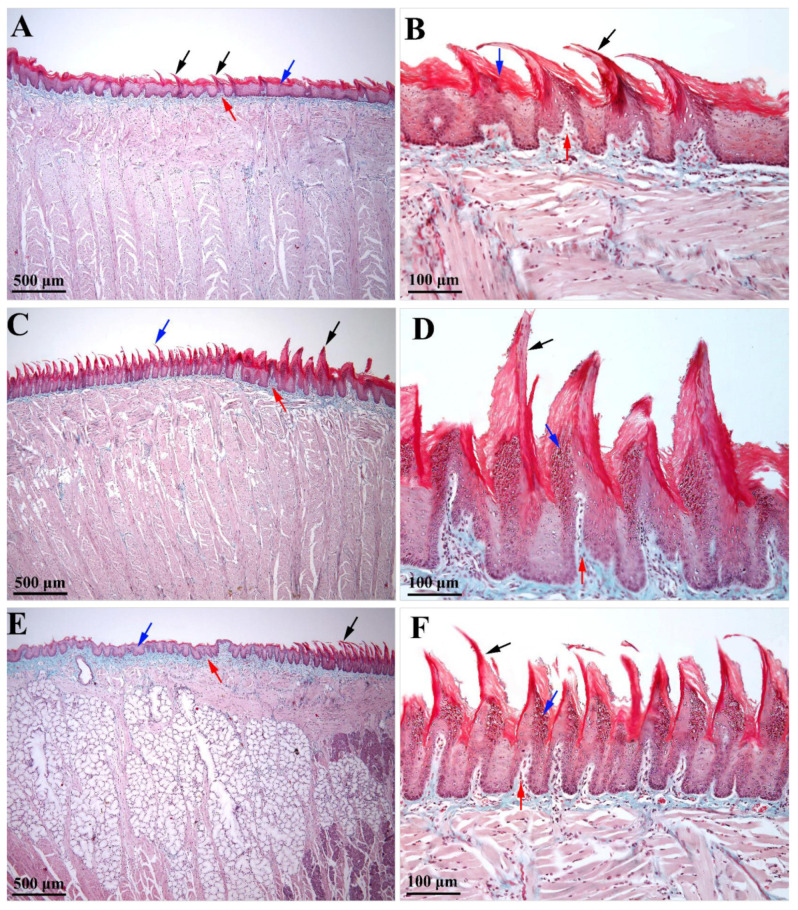
Microscopical features of the tongue of a white Wistar rat (Goldner’s trichrome stain): (**A**) anterior portion of the lingual body that presents short filliform papillae (black arrow); squamous stratified keratinized epithelium (blue arrow) disposed on lamina propria (red arrow); (**B**) the anteroposterior inclination of the filliform papillae (black arrow) from the anterior portion of the lingual body, with a thicker covering of keratinized squamous stratified epithelium on the anterior part of each filiform papilla (blue arrow) and subjacent lamina propria (red arrow); (**C**) the orientation of the filliform papillae from the posterior portion of the lingual body (blue arrow) compared to the ones from the lingual protuberance (black arrow), which are sustained by a thicker lamina propria (red arrow); (**D**) lingual protuberance with a distinct posteroanterior-inclined conical filliform papillae (black arrow) which have a prominent granular layer highly charged with keratohyalin granules on the posterior face of the papillae (blue arrow) vs. the anterior face; red arrow suggests the sustaining connective tissue of the filliform papillae; (**E**) crossing area from the posterior portion of the lingual body (black arrow) and the root of the tongue (blue arrow), the last one displaying only some elevation of covering the epithelium on the sustaining lamina propria (red arrow); (**F**) details of the posterior portion of the lingual body that includes anterioposteriorly oriented filliform papillae (black arrow) with a prominent granular layer (blue arrow) on the anterior face of each papilla; sustaining lamina propria protruding in the central ax of the filliform papillae (red arrow).

The density of the filiform papillae on the lingual apex is 86.25 per 1 mm^2^. However, on the lingual body, the status is different in the anterior part (i.e., between the apex and the protuberance), as compared to the posterior part (i.e., between the protuberance and the root). On the anterior part of the lingual body (Figure 1C and Figure 2A,B), the filiform papillae are slightly higher than on the apex; they have an anteroposterior curvature, similar to those on the apex, but their density is slightly lower, with an average of 82 per 1 mm^2^.

On the tongue protuberance (Figure 1D and Figure 2C,D), about 10 rows of papillae were identified as significantly higher and thicker than the other regions of the tongue, with slightly posteroanterior-inclined conical tips. In this region, the lamina propria was significantly thicker than in the previous region (Figure 2C). The average density of these giant papillae on the protuberance was 36.6 per 1 mm^2^. The posterior part of the body of the tongue (Figure 1E and Figure 2E,F) was covered with papillae slightly shorter than those on the protuberance, but significantly longer than those on the apex and the anterior part of the lingual body. These papillae were inclined anteroposteriorly, but the degree of inclination was lower than in the papillae on the apex and the anterior part of the lingual body. These papillae were very numerous; being placed next to each other, their density was the highest of all of the regions of the tongue, with an average of 135.25 per 1 mm^2^. Particular to these papillae is the fact that their tip is not conical but branched. In the area between the body and the root, the filiform papillae visibly decrease in height, so that at the level of the body there are no more true papillae, but only slight elevations of the lingual epithelium (Figure 2E).

The epithelium on the dorsal face of the white Wistar rat tongue is an intensely keratinized stratified squamous epithelium. The epithelium covering the filiform papillae has the highest degree of keratinization, but keratinization is also present in the epithelium placed in between the papillae so that the entire epithelium on the dorsal surface of the tongue is intensely keratinized. Additionally, this species has a medium degree of keratinization of the epithelium on the ventral side of the tongue.

Some differences were detected in the covering epithelium of the filiform papillae from the anterior part of the tongue, as compared to the posterior one. Thus, the epithelium of the anteroposteriorly inclined filiform papillae of the anterior part of the tongue (i.e., the apex and body regions) was thicker and featured a thicker layer of keratin (Figure 2B). Moreover, the epithelium on the anterior part of each filiform papilla included several rows of cells in the granular layer whose cytoplasm was loaded with prominent keratohyalin granules. Oppositely, in the epithelium from the posterior part of the papillae, the granular layer did not include prominent intracytoplasmic keratohyalin granules. The presence of several rows of cells with keratohyalin granules only in the anterior epithelium of the filiform papillae could suggest that the anterior face of the papillae is extensively exposed to mechanical stimuli (during mastication–prehension processes), as compared to the posterior papillary epithelium. Consequently, in the anterior epithelium of the filiform papillae, the rate of cell replacement was higher than in the posterior one, a fact that could be responsible for the shape and curvature of such papillae. However, a variation was identified in the epithelium of the filiform papillae from the lingual protuberance (i.e., the papillae that were inclined in the posteroanterior direction). In these papillae, the posterior epithelium had a prominent granular layer that was highly charged with keratohyalin granules (Figure 2D). The morphological features of the filiform papillae from the lingual protuberance may suggest that the rear part of these papillae was more exposed to mechanical stimuli than the front, at least during the process of grinding the food, due to being in contact with the hard palate. Due to their size and their posteroanterior inclination, these papillae may play some other obscure roles, in addition to those played by the regularly oriented filiform papillae from the apex and lingual body.

Some anatomical features described in this paper on the dorsal surface of the tongue in the white Wistar rat are similar to the aspects found in other rat species. Thus, Davydova et al. [15] found that the white laboratory rat has a very prominent dorsal lingual protuberance, preceded by a lingual fossa and a prominent median groove (sulcus) about 1.4 cm long, a size that has also been found in the African giant rat [16]. The Nile grass rat has a dorsal lingual protuberance with a bifurcated apex, while the long-eared hedgehog has only a small elevation [5]. Lingual protuberance is present in several species of mammals, and especially in those that eat fibrous vegetation, such as grass, tubers, and roots, of which we mention most rodents and caviidae [17].

The lingual protuberance (*Torus linguae*) is a muscle formation present in most herbivorous mammals, including goats, cattle, and alpacas. According to some authors, this protuberance with muscular structure is considered to be a place for ruminants to direct food laterally so it can be crushed by the molars and palatal rugae on the surface of the protuberance [8]. However, herbivores are not the only mammals that have this formation; it also exists in other species such as the Egyptian bat (*Pipistrellus kuhlii*), which is insectivorous [18]. It is present in rodents such as guinea pigs, blind slippery rats, WWCPS rats, lesser bamboo rats, and large bamboo rats [8,17,19]. There are also exceptions such as the Persian squirrel that lacks lingual protuberance [20]. The role of this lingual structure rich in filiform papillae is to facilitate the chewing of food in the oral cavity by crushing it between the tongue and the hard palate [10], especially food rich in cellulose [16,18].

The surface of the tongue includes lingual papillae, whose distribution, morphology and density vary among species depending on food requirements and types, as well as environmental conditions [1]. The papillae are present on the surface of the tongue in the vast majority of mammalian species, but there are exceptions such as the pangolin in which the tongue is lacking papillae, which may suggest that its tongue is not specialized for food handling in the oral cavity and taste perception, but it is primarily used to catch insects with their sticky surface and transport them to the following segments [21]. In our study, it was found that, in Wistar rats, the papillae are present on the dorsal surface of the apex and lingual body, and extended on the edges, but are missing on the root and ventral surface. By comparison, the lingual papillae are scattered on the dorsal surface of the tongue epithelium of the Nile grass rat (*Arvicanthis niloticus*) and the Egyptian long-eared hedgehog (*Hemiechinus auritus*), but are missing on the ventral surface in both species [5]. In white laboratory rats, the filiform papillae are present on the tip, dorsal and lateral surfaces of the tongue; being densely distributed on the dorsal surface, the distance between them increases from the tip of the tongue to its root, and the papillae become lower and wider [15]. In the African giant pouched rats, Igbokwe and Mbajiorgu [16] found that filiform papillae were distributed on the dorsal and ventral sides of the apex, on the main body, on the lingual prominence, and on the root.

However, some peculiarities regarding the distribution of lingual filiform papillae have been described in some other species. By conducting comparative studies on the tongues of three species of camelidae, some authors have concluded that there are differences in the distribution of filiform papillae at the level of the lingual apex. Thus, in guanaco (*Lama guanicoe*), the filiform papillae are present in large numbers at the level of the lingual apex and the anterior part of the lingual body. Llamas (*Lama glama*) do not present true papillae at the level of the lingual apex but only on the lingual body, and alpacas (*Vicugna pacos*) have long, thin filiform papillae, directed caudally only at the level of the lingual groove and not on the rest of the dorsal surface of the lingual apex [22]. In the case of the greater Japanese shrew-mole, there are numerous filiform papillae distributed on the entire dorsal surface of the tongue, except for the lingual radix [23], a situation that is also found in the European mole [24]. In cats and bats, the lingual filiform papillae are present on the entire dorsal surface [9,21]. As a detail, the density of lingual papillae in feral cats is similar to that of Egyptian cats [9]. In the lesser bamboo rat, the filiform papillae are present in large numbers on the entire lingual surface, except for the radix and the posterior third of the ventral surface [8]. In rabbits, the filiform papillae are concentrated on the caudal part of the tip of the tongue [9].

The mode of feeding in young animals was associated with a special distribution of lingual filiform papillae. Thus, kittens have marginal papillae on the anterolateral edges of the tongue that help both to hold the nipple and to prevent milk from escaping through the space between the tongue and the hard palate. The shape of these lingual papillae changes significantly as cats grow and become adults [9].

Mechanical papillae, such as the filiform ones, show great diversity among mammal species [25]. In the rabbit and the Persian squirrel there was only one type of filiform papillae observed [26,27], while in the African giant pouched rat there were four types of filiform papillae identified: the first type had a long pointed process, the second had a robust base (on the lingual prominence), and the third was conically shaped with a pointed process, whereas the fourth type had a branched filamentous process (the filiform papillae on the lingual radix) [16].

In the case of the Wistar rat, three types of filiform papillae have been highlighted, arranged in three different areas: the apex, protuberance, and the lingual body. Those on the dorsal surface of the apex are relatively short filiform papillae with a sharp tip and are significantly inclined anteroposteriorly (claw-shaped). Such papillae are also present on the anterior part of the lingual body, with the difference that they are slightly higher there. On the protuberance, there are several rows of very high papillae with the tip oriented anteriorly, meaning exactly the opposite of the other papillae. Their shape is somewhat intermediate, between filiform and conical, so some authors call them conical and others filiform. The third type is present on the back of the lingual body: these are medium-sized filiform papillae with a branched apex, inclined anteroposteriorly (such as those on the apex), but the degree of inclination is slightly lower than in the case of those on the apex.

The density of the papillae on the surface of the tongue of the Wistar rat differs from one region of the tongue to another (Table 1), the highest density being recorded on the posterior portion of the lingual body (135.25/mm^2^) and the lowest density on the lingual protuberance (36.6/mm^2^). By comparison, the density of filiform papillae in the European mole is about 160/mm^2^ at the apex, and on the body of the tongue the density increases, ranging from 240 to 270/mm^2^ [24]. The average density of filiform papillae on the rabbit tongue was 81/mm^2^ on the lingual apex, 56.25/mm^2^ on the lingual body, and 36/mm^2^ on the root of the tongue [26]. However, in our report, the height of the lingual filiform papillae in Wistar rats varied from one region of the tongue to the others. The highest filiform papillae were recorded on the lingual protuberance (480.65 ± 32.06 μm) and the lowest on the lingual apex (168.16 ± 26.17 μm; Table 1). The height of the filiform papillae in African giant pouched rats varied from 344.24 ± 20.54 μm to 563.41 ± 25.12 μm [16]. In the white laboratory rat, Davydova et al. [15] found that the filiform papillae are dense, with a height of 368.67 ± 15.96 μm on the tip of the tongue and 323.36 ± 6.48 μm on the body of the tongue. The conical filiform papillae are located near the root of the tongue, being arranged at a greater distance from each other than the anterior papillae, and have a height of 272.53 ± 3.85 μm [15]. In the domestic rabbit, the average height of the filiform papillae is 554 μm on the lingual apex, 650 μm on the lingual body, and 532 μm on the root of the tongue [26]. In adult aardvark (*Orycteropus afer*), the value recorded for the height of filiform papillae present on the body of the tongue was 379.30 ± 63.95 µm vs. 483.02 ± 47.03 μm tall on the caudal part of the tongue [20].

Due to their height, and especially their strongly curved anteroposterior shape, the filiform papillae on the lingual apex appear to participate primarily in the retention of food. The papillae on the lingual body seem to be involved in retaining food particles on the surface of the tongue, but at the same time ensure that the food is directed towards the pharynx [10]. The filiform papillae are inclined anteroposteriorly in many animal species (e.g., goat, buffalo, barking deer, alpaca, etc.), but not in the case of goitered gazelle, where the filiform papillae are irregularly inclined [10].

The posteroanterior orientation of the lingual papillae on the protuberance in the Wistar rat most likely suggests that they perform the function of directing the food bolus on the surface of the protuberance and the grinding of food when it is between the papillae and the hard palate [16]. Another possible function of these robust papillae is the sorting of foreign bodies from ingested food to reject them from the oral cavity, or they could play a role in saliva distribution inside of the oral cavity to ease grooming. Aspects regarding the orientation and shape of the filiform papillae have been reported by other authors. Thus, in the African giant pouched rat, all types of filiform papillae were directed caudally towards the root, except for those on the lingual protuberance [16]. Some authors state that the papillae on the protuberance are filiform and directed perpendicularly such as in the Middle East blind mole rat [28], while others describe them in rats as large conical papillae [29]. The papillae on the lingual protuberance of the European mole were also named conical papillae, with the specification that they were anteriorly inclined [24]. Some authors claim that the papillae on the lingual protuberance of the white laboratory rat are massive and wide foliate papillae [15]. In the lesser bamboo rat, there are anterovertically oriented filiform papillae on the anterior part of the lingual prominence. Those who described them believe that their role may be to help the bolus slide on the surface of the protuberance so that it can be moved laterally from one row of teeth to another for chewing [8]. In ruminants, lingual protuberance also plays an important role in the ruminating process [30].

Histologically, the dorsal surface of the tongue of the Wistar rat is covered by an intensely keratinized stratified epithelium. On the ventral side of the tongue, the epithelium is poorly keratinized. The degree of keratinization of the lingual covering epithelium is closely related to the type of food [1] and becomes thicker in areas such as the tip and body of the tongue that are frequently exposed to mechanical injuries and trauma. Comparing the degree of keratinization of the lingual epithelium in rats and fruit bats, Abayomi et al. [21] found intense keratinization in rats that eat hard food compared to the frugivorous bat that has low keratinization levels because it feeds on juicy ripe fruit. Massoud and Abumandour [5] also found differences between the Nile grass rat, which showed a higher degree of keratinization, and the Egyptian long-eared hedgehog, in which keratinization was significantly lower.

## 4. Conclusions

The study of macro and microanatomy in mammals is important since it helps in the assessment and understanding of external features of species, including behavior. Moreover, the addition of new morphological features may help to determine some evolutionary details of the investigated species. Our paper draws attention to new morphological details of the tongue of the Wistar rat, a species that is extensively utilized and crucial in biomedical research, as is the use of rat tongue [31,32,33]. The wealth of information available on the rat is undeniable, but new structural details are always welcome to the so-called “best functionally characterized mammalian model system” [34].

## Figures and Tables

**Table 1:** The features of filiform papillae in all the regions of the tongue.

Area	Average Papillary Density	Average Papillae Height (μm)	Bending and Orientation of the Papillae	Descriptive Features of the Papillae
Lingual apex	86.3/mm^2^	168.2 ± 26.2	anteroposterior	- Shortest of all tongue regions - A sharp tip
Lingual body (anterior portion)	62/mm^2^	248.5 ± 30.5	anteroposterior	- Slightly taller vs. the apex - Lower density vs. the apex/posterior lingual body
Lingual body (protuberance)	36.6/mm^2^	480.7 ± 32.1	posteroanterior	- Significantly taller than all the other papillae - Thickest of all tongue regions - Lowest density of all tongue regions
Lingual body (posterior portion)	135.3/mm^2^	341.4 ± 22.1	anteroposterior	- Slightly shorter vs. protuberance papillae - Degree of bending is lower vs. the other anteroposteriorly oriented papillae - The highest density of all tongue regions - tip is branched (not conical)

## Data Availability

The data presented in this study are available in the article.
